# Autophagy in Neurodegenerative Diseases: A Hunter for Aggregates

**DOI:** 10.3390/ijms21093369

**Published:** 2020-05-10

**Authors:** Hyungsun Park, Ju-Hee Kang, Seongju Lee

**Affiliations:** 1 Department of Anatomy, College of Medicine, Inha University, Incheon 22212, Korea; hyungsun@inha.edu; 2 Hypoxia-related Disease Research Center, College of Medicine, Inha University, Incheon 22212, Korea; johykang@inha.ac.kr; 3 Department of Pharmacology, College of Medicine, Inha University, Incheon 22212, Korea

**Keywords:** autophagy, neurodegenerative disease, protein aggregates

## Abstract

Cells have developed elaborate quality-control mechanisms for proteins and organelles to maintain cellular homeostasis. Such quality-control mechanisms are maintained by conformational folding via molecular chaperones and by degradation through the ubiquitin-proteasome or autophagy-lysosome system. Accumulating evidence suggests that impaired autophagy contributes to the accumulation of intracellular inclusion bodies consisting of misfolded proteins, which is a hallmark of most neurodegenerative diseases. In addition, genetic mutations in core autophagy-related genes have been reported to be linked to neurodegenerative diseases, such as Alzheimer’s disease, Parkinson’s disease, and Huntington’s disease. Conversely, the pathogenic proteins, such as amyloid β and α-synuclein, are detrimental to the autophagy pathway. Here, we review the recent advances in understanding the relationship between autophagic defects and the pathogenesis of neurodegenerative diseases and suggest autophagy induction as a promising strategy for the treatment of these conditions.

## 1. Autophagy Process

Autophagy is an evolutionary conserved intracellular degradation process. Mammals present three types of autophagic processes according to the adopted mechanism of cargo transport into lysosomes: chaperone-mediated autophagy, microautophagy, and macroautophagy, hereafter simply referred to as autophagy. Unnecessary or misfolded proteins and damaged subcellular organelles are engulfed by autophagy-specific double membrane-bound vesicles, called autophagosomes, and delivered to the lysosomes for breakdown. In almost all cells, autophagy occurs at a low basal level under inhibition by the mammalian target of rapamycin complex 1 (mTORC1), a key regulator of autophagy, to sustain cellular homeostasis. Upon various types of cellular stress, such as nutrient deprivation, growth factor withdrawal, or hypoxia, autophagy is released from mTORC1 inhibition and is highly upregulated to meet high energy demands.

### 1.1. Initiation of Autophagy

**Figure 1:**
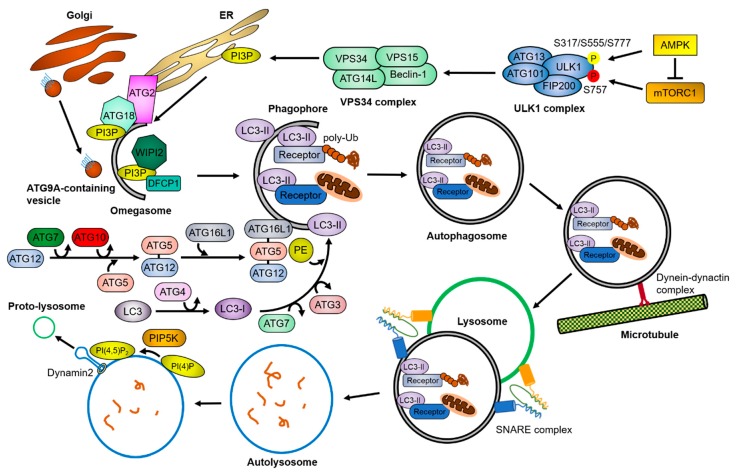
Overview of the autophagy pathway. Upon cellular stresses, mTORC1 and AMPK phosphorylates the ULK1 complex and the VPS34 complex sequentially (P in yellow circle, activation; P in red circle, inhibition). The VPS34 complex generates PI3P in lipid-enriched membranes, which recruit PI3P-binding proteins. This lipid–protein complex facilitates the assembly of autophagy-related proteins, resulting in the formation of omegasomes. During the growth of the omegasomes to the autophagosomes, the ubiquitin-like conjugation systems of ATG proteins convert LC3 to LC3-II by conjugating PE, and the autophagic receptors and adaptor proteins transport cargoes to the autophagosomes. The matured autophagosomes are transported near the lysosomes by a dynein-dynactin complex, where they form the autolysosomes through the SNARE complex. After the fusion, lysosomal proteases degrade the cargoes. Meanwhile, PI(4,5)P_2_ builds lysosomal tubule formation sites, generating the proto-lysosomes required for maintaining the free-lysosome pool in the cytosol. The detailed mechanisms are described in Section 1.

The autophagy process (summarized in Figure 1) consists of a series of molecular events, each of which is managed by autophagy-specific complexes whose activity is tightly regulated by upstream signaling molecules. Two main signaling molecules of autophagy, mTORC1 and AMP-activated protein kinase (AMPK), conversely regulate autophagy activity by phosphorylating Unc-51 like autophagy activating kinase 1 (ULK1) at different sites. mTORC1 inhibits autophagy initiation by phosphorylating ULK1 at Ser757, whereas AMPK promotes autophagy initiation by phosphorylating ULK1 at Ser317, Ser555, and Ser777 [1,2,3]. The ULK1 complex, known as an autophagy preinitiation complex, is composed of the ULK1 protein kinase, FIP200/RB1CC1, and the regulatory subunits ATG101 and ATG13, which are known to activate ULK1 through conformational changes [4,5]. The activated ULK1 complex phosphorylates a downstream autophagy initiation complex, the class III phosphoinositide 3-kinase (PI3K) VPS34 complex, which is composed of Beclin-1, VPS34, VPS15, and ATG14L [6,7,8,9]. The VPS34 complex generates phosphatidylinositol 3-phosphate (PI3P) in specialized phospholipid membranes, such as the endoplasmic reticulum (ER), ER-mitochondria contact sites, and ER-plasma membrane contact sites [10,11,12]. PI3P-binding proteins, such as zinc finger FYVE domain-containing protein 1 (ZFYVE1)/DFCP1 or WD repeat domain phosphoinositide-interacting proteins (WIPIs), are recruited to the PI3P-enriched membrane structures, called omegasomes, and further recruit autophagy-related proteins to generate the phagophore structure [13,14].

### 1.2. Autophagosome Formation

Two ubiquitin-like conjugation systems toward ATG8s (LC3A/B/C, GABARAP, GABARAPL1, and GABARAPL2 in mammals) are required for elongation of the phagophore. In the first system, ATG12 is covalently linked to ATG5 through the ATG7- and ATG10-mediated ubiquitin-like activating pathway [15]. ATG16L1 non-covalently binds to ATG5 through the interaction of coiled-coil domains and forms the ATG12–ATG5–ATG16L1 complex, which acts like the E3 ubiquitin ligase on the phagophore [16,17]. In the second system, the protease ATG4 cleaves the C-terminal region of LC3 to produce LC3-I into the cytosol [18,19]. Next, LC3-I undergoes a cascade reaction of ATG7, ATG3, and ATG12–ATG5–ATG16L1, resulting in the formation of LC3-II conjugated with phosphatidylethanolamine (PE) [20]. LC3-II is tightly associated with the double-membrane phagophore during the elongation period [21,22].

Many recent studies have revealed the detailed regulatory mechanisms of phagophore expansion. ATG9A is the only transmembrane protein of the autophagy core proteins. When autophagy is activated, ATG9A is transferred from the trans-Golgi network (TGN) or the endocytic compartments to the omegasomes in a ULK1- or retromer complex-dependent manner [23,24,25]. The translocation of ATG9A-containing single-membrane vesicles to the outer membrane of autophagosomes is a crucial step for autophagosome formation, suggesting that the ATG9A-containing vesicles act as lipid bilayer donors in this process [26,27,28,29]. Another ATG protein, ATG2, is associated with the phagophore through the PI3P binding ability of ATG18 [30,31,32]. The ATG2-ATG18 complex tethers the PI3P lipid-enriched membrane to the phagophore, which is required to control the size of the early autophagic structures [33,34,35]. Furthermore, the yeast Atg2 protein was reported to possess a lipid transfer activity, which is essential for autophagy [36]. Although the precise mechanisms of autophagosome closure are still not clear, accumulating evidence suggests that ATG2, VPS21, and the endosomal sorting complexes required for transport (ESCRT) complex play a role in autophagosome closure [35,37,38,39].

### 1.3. Autophagosome Maturation

To fuse with lysosomes, autophagosomes move toward them through the intracellular trafficking systems. The dynein–dynactin motor complex and Rab proteins on the microtubules are necessary for autophagosome movement to lysosome-concentrated regions [40,41]. Particularly in neurons, most autophagosomes are generated in the axon tip and undergo a dynein-mediated retrograde transport to the soma [42,43,44]. Meanwhile, lysosomes also migrate toward autophagosomes in a GTPase- or retromer-dependent manner [45,46,47]. Finally, autophagosomes fuse with lysosomes to form autolysosomes through the soluble N-ethylmaleimide-sensitive-factor attachment protein receptor (SNARE) complex [41,48,49]. At the end of the autophagy process, clathrin-mediated lysosomal tubular structures are extended from the autolysosomes, referred to as autophagic–lysosomal reformation (ALR) [50]. PI(4,5)P_2_ generated by the phosphatidylinositol-4-phosphate 5-kinase (PIP5K) is an essential factor for the initiation of autolysosome tubulation [51]. The large GTPase dynamin 2 is also involved in the scission of the lysosomal tubules during ALR [52,53].

### 1.4. Selective Autophagy

Initially, autophagy was thought of as a nonselective, bulk degradation process [54]. However, most autophagy is now regarded as a selective degradation process, as many kinds of autophagic receptors have been identified [55,56,57]. Autophagic receptors recognize and recruit specific cargoes to the autophagosome, for example, p62 for protein aggregates and nuclear FMR1 interacting protein 1 (NUFIP1) for ribosomes. Depending on the target molecules, selective autophagy is divided into aggrephagy for protein aggregates, mitophagy for mitochondria, ER-phagy for ER, pexophagy for peroxisomes, xenophagy for pathogens, lipophagy for liposomes, lysophagy for lysosomes, proteaphagy for proteasomes, ribophagy for ribosomes, and granulophagy for granules [58]. Autophagic receptors mostly recognize target molecules via their conjugated poly-ubiquitin chains through a specific domain, such as the ubiquitin-associated (UBA) domain on p62 or NBR1 and the zinc finger (ZF) domain on OPTN [59,60,61,62]. After this recognition occurs, the autophagic receptors transfer the cargoes to autophagosomes by directly binding with ATG8s through the LC3-interacting region (LIR) or the GABARAP interaction motif (GIM) [63,64].

Growing evidence has connected the genetic variants of the autophagic receptors to various neurodegenerative diseases. Since the accumulation of protein aggregates is seen in most neurodegenerative diseases, it is plausible that the malfunction of the autophagic receptors, rather than other autophagy-related proteins, might be related to the pathogenesis of neurodegenerative diseases. The relationships between various autophagic receptors and neurodegenerative diseases are described in each section and summarized in Table 1.

## 2. Autophagy in the Nervous System and Neurodegenerative Diseases

### 2.1. Roles of Autophagy in the Nervous System

Autophagy is a lysosomal degradation pathway, which contributes not only to provide nutrients but also to remove harmful materials, such as misfolded proteins and invading microorganisms, to support cellular homeostasis and survival. Thus, defects in autophagy have been associated with various human diseases, including cancer, metabolic disorders, and neurodegenerative diseases. Hereafter, we will focus on the relationship between autophagic defects and neurodegenerative diseases.

Neurons are believed to be particularly vulnerable to malfunction of autophagy. Neurons are polarized cells with large amounts of cytoplasm and spatial heterogeneous endosomal populations; thus, the accumulation of cellular waste would represent a heavy burden for these cells [90,91]. The cellular waste also does not get diluted, since neurons do not divide. Indeed, knockout of *Atg* genes in mice led to embryonic or neonatal lethality, suggesting the indispensability of autophagy and generation of brain-specific knockout model systems to investigate the role of autophagy in the brain [92,93]. Knockout of the core autophagy protein, ATG5 or ATG7, in mouse neurons caused accumulation of polyubiquitinated inclusion bodies and behavioral defects [94,95]. Similarly, Purkinje cell-specific knockout of ATG5 or ATG7 induced accumulation of autophagosome-like double-membrane structures in axonal swelling, followed by deficits in motor function [96,97]. Depletion of another core autophagy protein, FIP200, in mouse neurons resulted in a reduction of autophagosome formation and cerebellar degeneration with progressive neuronal loss [98]. Silencing of p62, an autophagic receptor that specifically recognizes polyubiquitinated proteins, increased the formation of neurofibrillary tangles and behavioral abnormalities in mice and zebrafish [66,99]. Conversely, overexpression of p62 in the mouse brain attenuated amyloid β (Aβ) pathology and ameliorated cognitive ability by upregulating autophagic clearance [65]. Recently, an unbiased genome-wide screening in the mouse striatum revealed that some ATG proteins, such as ATG5, ATG7, ATG101, and *ATG16L1*, are crucial for neuronal survival [100]. Those results indicate that autophagy is required to maintain neuronal homeostasis and remove protein aggregates, which are fundamental steps to prevent neuronal disorders.

### 2.2. Relationship of Autophagy Pathway with Pathogenesis of Neurodegenerative Diseases

**Figure 2:**
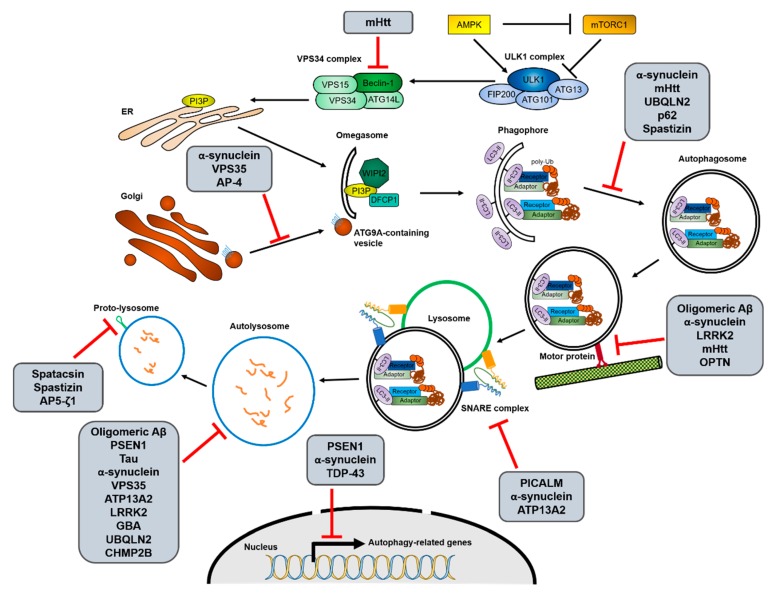
Interactions between the autophagy process and neurodegenerative disease-associated proteins. Increasing evidence for interactions between autophagy and neurodegenerative diseases suggests that not only the decrease of autophagic activity is one of causes of the diseases, but mutations in disease-associated genes also inhibit autophagy in various stages. The possible interruptions in each autophagic process from initiation to reformation of free lysosomes by the different neurodegenerative disease-associated mutant proteins are shown. The detailed relationships between the mutant proteins and autophagy pathway are described in Section 2.

Most neurodegenerative diseases exhibit pathological abnormal protein aggregates, developing neurofibrillary tangles; for example, Aβ and C-terminal fragments (CTF) of the amyloid precursor protein (APP) in Alzheimer’s disease (AD), mutant α-synuclein in Parkinson’s disease (PD), polyglutamine (polyQ)-expanded huntingtin (mHtt) in Huntington’s disease (HD), and mutant superoxide dismutase 1 (SOD1) and TAR DNA-binding protein 43 (TDP-43) in amyotrophic lateral sclerosis (ALS) [57,101,102]. These protein aggregates observed in neurodegenerative diseases are mainly targeted towards the autophagy–lysosome degradation pathway. Similarly, genetic mutations in autophagic receptors, such as p62, OPTN, NBR1, and ALFY/WDFY3, have often been associated with neurodegenerative diseases [56,57]. Aging, the most common risk factor for neurodegeneration, also significantly decreases autophagic activity [103]. Therefore, it is thought that dysfunctional autophagy can lead to the development of neurodegenerative diseases. Recently, various studies have suggested that the modulation of autophagy could be a promising strategy for treating these conditions. The clearance of aggregate-prone proteins, such as mHtt, insoluble tau, and Aβ42, was increased by the activation of autophagy and dependent on the aggrephagy receptor, p62 [65,75,104,105,106]. Conversely, inhibition of autophagy using 3-MA or bafilomycin A1 (Baf. A1) increased mHtt aggregates in cell culture systems and rat brains [106,107]. Here, we review the recent advances in understanding the pathogenesis of neurodegenerative diseases in relation to autophagy (summarized in Figure 2) and discuss autophagy modulation as a therapeutic strategy for these diseases.

#### 2.2.1. Alzheimer’s Disease (AD)

AD is the most common neurodegenerative disease, causing dementia. Accumulation of Aβ plaques and tau neurofibrillary tangles in the brain of AD patients is considered a pathological characteristic of the disease, as well as a crucial part of its pathogenesis. Aβ, a central component of amyloid plaques, is a small peptide derived from the sequential processing of APP. APP is mainly cleaved into the TGN and the endosomes by sequential activation of α-, β- and γ-secretase [108,109]. The clearance of Aβ and APP–CTF is mainly achieved through autophagy [110,111,112]. The increase in the activity of p62 or transcription factor EB (TFEB) was shown to attenuate Aβ plaque formation, resulting in the improvement of AD pathology in mice [65,113]. Contrastingly, increased Aβ oligomers hindered autophagic activity through the impairment of trafficking and lysosome biogenesis in animal models [114,115]. The ultrastructural analysis of the brain of AD patients revealed that cathepsin-containing autolysosomes accumulated due to defects of lysosomal proteolysis [116,117,118]. Furthermore, the levels of autophagy-related proteins are frequently altered in AD patients’ samples [119,120,121]. The genes linking autophagy to AD are discussed in detail below.

Some mutations causing familial AD are reported to affect autophagy. Presenilin 1 (PSEN1/PS-1) is a component of the γ-secretase complex that generates Aβ peptide [122]. To date, a number of genetic mutations of PSEN1 have been found to affect APP processing and to be linked with AD pathogenesis [123]. Several loss-of-function analyses have revealed that PSEN1 is required for lysosomal homeostasis and transcription of autophagy-related genes [124,125,126]. PSEN1 mutations causing familial AD lead to aberrant v-ATPase trafficking to lysosomes, thus resulting in lysosomal alkalization and accumulation of dysfunctional autolysosomes [126,127,128]. PSEN1-deficient neural stem cells also exhibited reduced TFEB expression, leading to decreased transcription levels of autophagy-related genes [125].

Various single nucleotide polymorphisms (SNPs) and abnormal cleaved forms of phosphatidylinositol-binding clathrin assembly protein (PICALM) have been reported in AD [129,130]. PICALM is a clathrin adaptor protein involved in the clathrin-mediated endocytosis of SNARE proteins and APP. PICALM and APP colocalize during endocytosis, and genetic downregulation of PICALM selectively decreases endocytosis of APP and Aβ plaque generation in the brain of mice [71]. Recent studies have shown that PICALM is involved in multiple steps of the autophagic pathway, regardless of its role in APP endocytosis. Indeed, PICALM affects the formation and maturation of autophagosomes by regulating endocytosis of SNAREs, such as VAMP2, VAMP3, and VAMP8 [131]. SNAREs are known to mediate the fusion of autophagosomes with lysosomes [49]. Additionally, PICALM functions as an autophagic receptor and makes a complex with adaptor protein 2 (AP-2). The AP2-PICALM complex bridges the APP-CTF and LC3B for autophagic degradation [72].

The decreased autophagy activity in AD patients may also be attributed to Beclin-1 and VPS35, a core retromer component. Reduced levels of Beclin-1 and VPS35 have been reported in AD patients [132,133]. Genetic downregulation of Beclin-1 in mice resulted in reduced neuronal autophagy and Aβ accumulation, leading to neurodegeneration [133]. Mechanistically, Beclin-1 promotes APP trafficking from the cell surface to the autophagosomes by direct interaction with APP via an evolutionarily conserved domain (ECD) [134]. Interestingly, Beclin-1 regulates phagocytosis in neurons by regulating VPS35 protein levels [132]. Knockdown of VPS35 in culture cells has also been found to cause Aβ accumulation [135].

Degradation of aberrant phosphorylated tau also depends on autophagy [110,136]. Accumulation of proteins in the autophagy–lysosomal pathway, such as p62, LC3, and LAMP1, as well as autophagic and lysosomal defects were observed in the post-mortem brains of patients that had AD [67]. The protein aggregates were colocalized with hyperphosphorylated tau (S422) [67,68]. Hyperphosphorylated tau showed direct bindings with aggrephagy receptors, such as p62, NDP52, and OPTN, and was subjected to autophagic degradation [66,69,70]. PICALM also mediates autophagic degradation of tau [131]. Besides, impaired transport by the dynein–dynactin complex, which contributes to autophagosome trafficking, increased tau-positive filamentous inclusions [137]. Contrastingly, autophagy activation accelerated the degradation of phosphorylated tau, resulting in the inhibition of tau aggregation in vitro and in vivo [138,139].

#### 2.2.2. Parkinson’s Disease (PD)

PD is a progressive neurodegenerative movement disorder, characterized by pathological α-synuclein inclusions called Lewy bodies in the dopaminergic neurons of the substantia nigra and multiplication of the α-synuclein gene [140]. Knockout of ATG7 was shown to induce an age-dependent increase in the formation of p62-containing α-synuclein inclusion bodies in dopaminergic neurons and motor function deficits in aged mice [73]. Several studies indicate that α-synuclein bearing pathogenic mutations is degraded by the autophagy-lysosome system [141,142,143,144]. Conversely, α-synuclein inclusions impair the autophagic pathway in several steps. For instance, it was demonstrated that α-synuclein inclusions reduce omegasome formation by inducing the mislocalization of ATG9A [145,146]. Besides, α-synuclein aggregates damage the retrograde transport of autophagosomes, but not the autophagosome–lysosome fusion [146,147]. Finally, α-synuclein disrupts the activity of the lysosomal aspartyl protease cathepsin D (CTSD) and the autophagic degradation process [148,149,150].

Transcriptional alteration of autophagy-related genes is frequently observed in PD. During the symptomatic stage of PD, when most dopaminergic neurons in the midbrain are already lost, the TFEB-mediated transcription of Beclin-1, CTSD, and LAMP1 is decreased compared to the pre-symptomatic stage [151]. Moreover, the expression of human A30P α-synuclein increased the nuclear translocation of the transcriptional repressor zinc finger protein with KRAB and SCAN domains 3 (ZKSCAN3), which regulates the transcription of LC3 and p62 [152]. As expected, the pathological features of PD, including neurodegeneration in the midbrain, motor dysfunction, and α-synuclein accumulation, were improved by TFEB overexpression [153,154].

Mutations in glucocerebrosidase (GBA), a lysosomal enzyme degrading glucosylceramide, represent the most common genetic risk factor for PD. The PD-associated *GBA* mutations (N370S and L444P) reduce its protein levels and enzymatic activity and impair its trafficking from the ER to the lysosomes. This induces ER stress and the accumulation of target lipids in lysosomes, which eventually results in autophagy–lysosomal dysfunction [155,156,157]. Patients with sporadic PD show a selective decrease of GBA activity accompanied by increased α-synuclein inclusions at an early stage [158,159]. Direct inhibition or N370S mutation of GBA promotes the accumulation of α-synuclein oligomers [156,158,160]. Moreover, it was demonstrated that glucocerebroside, the GBA target lipid, can promote α-synuclein fibril formation; the lysosomal membrane-bound α-synuclein fibrils can then inhibit the activity and trafficking of GBA through direct binding, leading to further exacerbation of PD [161,162].

The next most common genetic risk factor in PD is represented by mutations in the leucine-rich repeat kinase 2 (LRRK2/PARK8) gene; more than 40 pathogenic *LRRK2* mutations have been reported in patients with PD [163]. However, it is still controversial whether the role of LRRK2 in autophagy is associated with PD pathology. Some studies have shown that LRRK2 loss impairs the autophagy–lysosome pathway, resulting in cell death [164,165]. Interestingly, many pathogenic mutations in *LRRK2* are gain-of-function mutations, for example, G2019S and R1441C [166]. The *LRRK2* gain-of-function mutations increase its kinase activity, but impair autophagic degradation, similar to LRRK2 deficiency [167,168,169,170]. This paradox would be explained by some studies showing that the LRRK2-G2019S mutant inhibits the endocytic vesicular trafficking by decreasing small GTPase activity and that the LRRK2–R1441C mutant inhibits lysosomal functions due to its defective binding to the lysosomal v-ATPase [171,172].

The loss-of-function mutants of ATPase cation transporting 13A2 (ATP13A2), which are characterized in an early-onset form of PD, were reported to be retained in the ER and not translocated to the lysosomes [173]. ATP13A2 is a lysosomal type 5 P-type ATPase, and therefore essential for the maintenance of the lysosomal pH. In PD patients, ATP13A2 protein levels were found to be decreased in dopaminergic neurons, and the existing low amounts of ATP132A2 proteins were located in the Lewy bodies [174]. Furthermore, the PD-associated mutations of *ATP13A2* were reported to cause impairment of lysosomal acidification [174]. Recent studies have shown the molecular mechanisms underlying the ATP13A2-mediated autophagy–lysosome pathway. Depletion of ATP13A2 induces the retention in the cytosol of TFEB, a critical transcription factor for autophagy-related genes, by regulating mTORC1 activity [175]. Moreover, the downregulation of Synaptotagmin 11 (SYT11) caused by ATP13A2 deficiency induces lysosomal dysfunction, leading to disruption of autophagic degradation [175]. Another study reported that ATP13A2 enhances autophagosome–lysosome fusion by facilitating HDAC6-dependent cortactin deacetylation that leads to the assembling of an F-actin network [176]. In addition, a mutation in *ATP13A2* was shown to cause α-synuclein accumulation and silencing of α-synuclein was able to attenuate the neurotoxicity induced by ATP13A2 depletion [177], suggesting that loss of ATP13A2 may contribute to PD pathology via α-synuclein accumulation.

The autophagy defects observed in PD may be partly attributed to the mutation of VPS35, a core retromer complex component, which has been reported to regulate trafficking of lysosomal protease [46,178]. mRNA levels of VPS35 were found to be decreased in the PD substantia nigra [179]. A familial PD-associated VPS35 mutation (D620N) was shown to impair the recruitment of the WASP and Scar homolog (WASH) complex to the endosomes, resulting in the mislocalization of ATG9A and defective autophagy [180].

#### 2.2.3. Huntington’s Disease (HD)

HD is an autosomal-dominant progressive neurodegenerative disease, exhibiting motor dysfunction, behavioral disturbances, and cognitive dysfunction. The abnormal expansion of a polyQ repeat in exon 1 produces mHtt proteins that form ubiquitin-positive aggregates with β-sheet rich structures, leading to cytotoxicity in the striatum and cortex [181,182]. The overexpression of mHtt induces progressive motor deficits accompanied by the accumulation of autophagosomes [183,184]. Similarly, the accumulation of autophagic vacuoles was observed in HD patients [185,186]. A number of studies have shown that autophagy clears aggregate-prone proteins with polyQ expansion from cell culture systems to in vivo [106,141,187,188,189]. In those studies, autophagy blockage by treatment with an autophagy inhibitor, such as 3-MA or Baf.A1, or genetic modulation increased mHtt aggregation. Contrastingly, an autophagy activator, such as rapamycin or trehalose, reduced the number of inclusion bodies. The turnover rate of mHtt varied depending on its structure, which influenced its interaction with the aggrephagy receptors p62 and OPTN [75,77,107]. The heterozygous deficiency of the aggrephagy adaptor ALFY, which facilitates the recruitment of the p62-binding intracellular inclusions to the autophagosomes, also promoted the aberrant accumulation of mHtt and HD progression in a mouse model [76,79]. A recent genome-wide screening in the striatum has suggested that many autophagy-related genes, such as *Atg4b*, *Tfeb*, and *Atlastin 3*, appear to prevent mHtt toxicity [100].

The normal huntingtin acts as a scaffolding protein for various autophagy proteins to facilitate cargo recognition and prevent Beclin-1 ubiquitination [190,191,192]. However, mHtt fails to recognize cytosolic cargoes in the autophagosomes [193]. mHtt was also shown to reduce the Beclin-1 activity by recruiting it to the inclusion bodies or inactivating a striatal specific GTPase, RASD family member 2 (RASD2/Rhes), which promotes the dissociation of inhibitory interactions between Beclin-1 and Bcl-2 [192,194]. Moreover, huntingtin knockdown or mHtt overexpression in neurons can lead to defective retrograde transport of the autophagosomes [42].

#### 2.2.4. Amyotrophic Lateral Sclerosis (ALS)

ALS is a rare progressive neurodegenerative disease, characterized by the death of motor neurons controlling the voluntary muscles as well as by cytoplasmic ubiquitin-positive inclusion formation. Most ALS cases are sporadic, but about 5% to 10% are caused by inherited genetic mutations in SOD1, TDP-43, fused in sarcoma (FUS), or ubiquilin 2 (UBQLN2) [195,196,197,198]. Many studies have suggested that autophagy might contribute to ALS pathogenesis. First, enlarged autophagosomes containing p62-positive aggregates were observed in ALS mouse models and ALS patients [80,199]. Secondly, motor neuron-specific ATG7 knockout mice bearing a *SOD1* pathogenic mutation have been found to have accelerated neuromuscular junction disruption as well as tremor, which are characteristics of ALS [199]. Thirdly, silencing of TDP-43 or one of its ALS-associated mutations increased the transcription of Bcl-2 and abnormal ATG4B proteins, resulting in autophagic defects [200,201,202]. Finally, autophagy activation reduced TDP-43 aggregates and improves the survival of human motor neurons bearing TDP-43 mutations [203].

UBQLN2, another genetic risk factor for familial ALS, has been associated with autophagy as well. UBQLN2 forms a complex with LC3 and ubiquitinates cargo proteins in the autophagosomes like the autophagy adaptor [204,205]. Moreover, UBQLN2 facilitates the autophagosome–lysosome fusion [204]. Loss of UBQLN2 was shown to increase ubiquitinated TDP-43 levels and impair autophagic degradation by promoting the fragmentation of lysosomal v-ATPase [89,206]. The overexpression of an ALS-associated UBQLN2 mutant, such as P497H, caused the formation of UBQLN2 inclusions bearing p62 and the accumulation of ubiquitinated-protein aggregates, resulting in defective autophagy and ALS-like phenotypes [85,86,207].

Recently, the correlation between ALS and aggrephagy receptor proteins, such as p62 and OPTN, has become more apparent. As mentioned above, TDP-43 inclusions were found to colocalize with p62, and to be degraded by p62 overexpression [81,82]. Conversely, p62 deficiency exacerbated ALS symptoms caused by the increase of insoluble protein aggregates [83,99]. Importantly, about half of the ALS-related mutations are located in the PB1, LIR, and UBA regions of p62 [57]. These regions are responsible for interacting with cargo proteins or LC3, suggesting that the ALS pathology originating from p62 mutations may be linked with the inefficient delivery of protein aggregates to the autophagosomes [84,99,208]. Another aggrephagy receptor, OPTN, promotes the clearance of protein aggregates by recognizing the inclusions through its coiled-coil domain [77]. OPTN silencing or its ALS-associated mutations increased the number of TDP-43- or SOD1-containing inclusions, resulting in necroptosis-dependent axonal degeneration [77,87,88]. In addition to its role as an aggrephagy receptor, OPTN contributes to autophagosome trafficking through MYO6 and TOM1 [209]. The interaction between OPTN and MYO6 is often disrupted in ALS patients or in case of mutations [78,210].

ALS can be also developed due to mutations in the ESCRT-III complex, which is important for autophagosome maturation. ALS-associated point mutations in CHMP2B, a subunit of the ESCRT-III complex, have been found to cause accumulation of autophagosomes and mislocalization of lysosomes, leading to the formation of intracellular inclusions in various model systems [211,212,213].

#### 2.2.5. Hereditary Spastic Paraplegia (HSP)

HSP is a diverse group of inherited neurodegenerative disorders characterized by axonal degeneration of the corticospinal motor neurons, leading to progressive weakness and spasticity of the legs. Until now, over 80 spastic gait (SPG) genetic loci have been reported, and more than 60 genes have been identified [214]. While various *SPG* genes exist, the molecular etiology underlying HSPs converges to a small number of cellular functions, such as membrane trafficking, mitochondrial function, organelle shaping and biogenesis, axonal transport, and lipid metabolism [215,216]. The most common form of autosomal-recessive HSP is induced by mutations in *SPG11* encoding spatacsin. Loss of spatacsin in mouse neurons has been found to induce aberrant lysosomal homeostasis, including the accumulation of autolysosomes and impairment of ALR [217,218]. Similar to spatacsin, depletion of spastizin, encoded by *SPG15*, diminished ALR initiation, resulting in reduced numbers of free lysosomes, and consequently in accumulation of cellular garbage [218]. Spastizin also regulates autophagosome maturation by interacting with the Beclin-1-UVRAG-Rubicon complex or by promoting Rab-dependent endosome–autophagosome fusion [219,220].

Several *SPG* genes encode adaptor protein (AP) complex subunits. The AP complex is located in the endosome or the TGN, and it is important for intracellular cargo sorting between distinct organelles. The AP-4 subunits β-1, μ-1, ε-1, and σ-1 are encoded by *SPG47*, *SPG50*, *SPG51*, and *SPG52*, respectively, and associated with childhood-onset HSP [221]. As the AP-4 complex mediates the sorting of the target proteins from the TGN to another membrane structure, AP-4 deficiency results in the missorting of ATG9A-containing vesicles from the TGN to autophagosome formation sites in the neurons [221,222]. Another AP complex subunit linked with HSP is AP-5 ζ-1, which is encoded by *SPG48*. A previous study reported that AP-5 interacts with spatacsin and spastizin [223]. Similar to the phenotypes obtained through silencing of spatacsin or spastizin, old AP-5 ζ-1 knockout mice exhibited degeneration of the corticospinal tract and incorporeal Golgi apparatus [224]. Furthermore, defects of autophagic clearance and ALR impairment can be observed in the AP-5 ζ-1 knockout mice.

## 3. Autophagy Upregulation as a Therapeutic Strategy for Neurodegenerative Diseases

**Figure 3:**
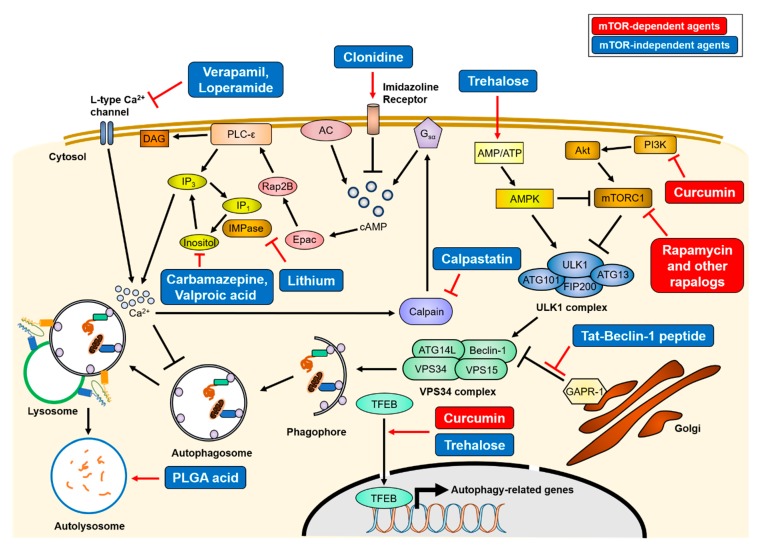
Mechanisms of therapeutics of autophagy-inducing agents. Treatment of various autophagy-inducing agents enhances the clearance of aggregate-prone proteins in an mTOR-dependent or -independent manner. The mTOR-dependent autophagy inducers (red), rapamycin and curcumin, directly inhibit mTORC1 activity, resulting in activation of the ULK1 complex. Conversely, mTOR-independent agents (blue) upregulate autophagy activity through various intracellular signaling cascade or lysosome biogenesis. The simplified actions of the agents are drawn, and detailed mechanisms are described in Section 3. AC = adenylyl cyclase, PLC-ε = phospholipase C-epsilon, DAG = diacylglycerol.

As mentioned above, various genetic mutations that reduce autophagic activity are linked to the pathology of inherited neurodegenerative diseases, and appear to aggravate them. Recent studies have shown that reducing the accumulation of intracellular aggregate-prone proteins by autophagy upregulation is beneficial to delay disease progression in AD, PD, HD, and ALS, suggesting that autophagy induction can be used as a therapeutic strategy for most neurodegenerative diseases [101,102,104,106,225,226,227] (summarized in Figure 3). However, extensive autophagy is detrimental to maintain cellular homeostasis. Therefore, caution should be taken when targeting autophagy for the treatment of neurodegenerative diseases.

### 3.1. mTOR-Dependent Autophagy Inducing Agents

Two types of small molecules can induce autophagy, in an mTOR-dependent or -independent manner. The first identified mTOR-dependent autophagy inducer is rapamycin. Rapamycin inhibits the kinase activity of mTORC1 by allosteric binding. Though not approved for clinical use, rapamycin treatment was shown to reduce neuronal death and improve most neurodegenerative disease symptoms in experimental settings by inducing autophagy [104,106,226,227]. Due to the limited absorption of rapamycin, its derivatives (defined rapalogs) have been developed, such as temsirolimus (CCI-779), everolimus (RAD001), and ridaforolimus (AP23575). Among them, everolimus has been recently approved by the Food and Drug Administration (FDA) for tuberous sclerosis treatment. In addition to rapamycin, curcumin, a natural compound that inhibits the PI3K/Akt/mTOR pathway, has been described as having a therapeutic effect on neurodegenerative diseases [228,229]. Curcumin has been shown to enhance the expression levels of autophagy-related proteins, such as Beclin-1, ATG5, or ATG16L1, and motor proteins essential for retrograde axonal transport, resulting in an increase of the autophagic flux and the clearance of intracellular aggregates [113,228,230,231].

### 3.2. mTOR-Independent Autophagy Inducing Agents

A long-term use of mTOR-dependent autophagy inducers could result in side effects, because the mTOR signaling has diverse autophagy-independent functions, such as ribosome biogenesis [102]. Therefore, mTOR-independent autophagy inducers have been developed. The representative mTOR-independent autophagy inducer is trehalose, which enhances autophagy by activating AMPK or TFEB [232,233]. Several studies have reported that this disaccharide promotes the clearance of protein aggregates and reduces neuronal death, thereby alleviating the progression of neurodegeneration diseases [138,141,148,234,235]. Some mood stabilizers, such as verapamil, loperamide, clonidine, and calpastatin, induce the degradation of aggregate-prone proteins by the autophagy-lysosome system via a reduction in inositol phosphate 3 (IP3) levels, which inhibits the autophagosome formation step. In the phosphoinositol cycle, lithium inhibits inositol monophosphatase (IMPase), whereas carbamazepine and valproic acid inhibit inositol synthesis [236,237].

Lysosomal alkalization by aberrant lysosomal ATPase causes autophagosome accumulation, resulting in the inhibition of the degradative pathway. Therefore, the re-acidification of lysosomes may be the proper strategy for autophagy enhancement. Restoring lysosomal pH using poly (DL-lactide-co-glycolide) (PLGA) acidic nanoparticles was shown to rescue lysosomal deficits and autophagic degradation [124,206].

Another therapeutic strategy involves small peptides. The 267–284 amino acid residues of Beclin-1 are known to be essential for autophagy induction [238]. The Tat-Beclin-1 peptide is a cell-permeable peptide of Beclin-1 (aa 267–284) conjugated with the HIV-1 Tat protein. The Tat-Beclin-1 enhances autophagy initiation by interacting with the autophagy inhibitor, Golgi-associated plant pathogenesis-related protein 1 (GAPR-1/GLIPR2), resulting in Beclin-1 distribution into the cytosol. This peptide also increases autophagosome formation in neurons. Besides, Tat-Beclin-1 has been shown to reduce mHtt aggregates in vitro.

## 4. Conclusions

Although inherited neurodegenerative diseases are caused by mutations in various genes, the accumulation of protein aggregates is a common characteristic they share. Thus, neurodegenerative diseases are considered proteinopathies. Numerous studies indicate that several genes associated with these conditions are involved in the autophagy–lysosome pathway and the intracellular protein aggregates can disrupt several steps of autophagy. Thus, we speculate that autophagy upregulation can ameliorate neurodegenerative diseases. In various experimental systems, autophagy activation reduces the accumulation of inclusion bodies and further alleviates neurodegeneration phenotypes. However, the intervention on neurodegenerative diseases using autophagy inducers is only in its infancy. The pharmacological autophagy modulation strategies currently in use are mostly based on the overall induction of the whole autophagy process. Besides, excessive autophagy activation results in toxic effects. Selective approaches should be implemented to target specific autophagy steps. To this end, it is necessary to thoroughly understand the roles of autophagy in various neurodegenerative diseases.

## Figures and Tables

**Table 1:** Association between various autophagic receptors/adaptors and neurodegenerative diseases.

Neurodegenerative Disease	Autophagic Receptor/Adaptor	Target Aggregates	Reference
Alzheimer’s disease	p62	Aβ	[65]
	p62	tau	[66,67,68,69]
	NDP52	tau	[70]
	OPTN	tau	[69]
	PICALM	Aβ, APP-CTF	[71,72]
Parkinson’s disease	p62	α-synuclein	[73,74]
Huntington’s disease	p62	mHtt	[75,76]
	OPTN	mHtt	[77,78]
	ALFY	mHtt	[76,79]
Amyotrophic lateral sclerosis	p62	TDP-43	[80,81,82]
	p62	SOD1	[83,84]
	p62	UBQLN2	[85,86]
	OPTN	TDP-43	[78,87]
	OPTN	SOD1	[77,87,88]
	UBQLN2	TDP-43	[89]
